# Association between semi-quantitative microbial load and respiratory symptoms among Thai military recruits: a prospective cohort study

**DOI:** 10.1186/s12879-018-3358-4

**Published:** 2018-09-14

**Authors:** Clarence C. Tam, Vittoria Offeddu, Kathryn B. Anderson, Alden L. Weg, Louis R. Macareo, Damon W. Ellison, Ram Rangsin, Stefan Fernandez, Robert V. Gibbons, In-Kyu Yoon, Sriluck Simasathien

**Affiliations:** 1 Saw Swee Hock School of Public Health, National University of Singapore and National University Health System, Singapore, 117549 Singapore; 2 London School of Hygiene & Tropical Medicine, WC1E7HT, London, UK; 3 University of Minnesota, Minneapolis, 55455 USA; 4 Armed Forces Research Institute of Medical Sciences, Bangkok, 10400 Thailand; 5 Phramongkutklao College of Medicine, Bangkok, 10400 Thailand; 6 University of Texas at San Antonio, San Antonio, 78249 USA; 7 International Vaccine Institute, Seoul, 08826 South Korea; 8 Phramongkutklao Hospital, Bangkok, 10400 Thailand

**Keywords:** Multiplex PCR diagnostics, Respiratory illness, Upper respiratory tract infection, Asymptomatic infection, Influenza-like illness, Influenza, *Haemophilus influenza*

## Abstract

**Background:**

Multiplex real-time polymerase chain reaction assays have improved diagnostic sensitivity for a wide range of pathogens. However, co-detection of multiple agents and bacterial colonization make it difficult to distinguish between asymptomatic infection or illness aetiology. We assessed whether semi-quantitative microbial load data can differentiate between symptomatic and asymptomatic states for common respiratory pathogens.

**Methods:**

We obtained throat and nasal swab samples from military trainees at two Thai Army barracks. Specimens were collected at the start and end of 10-week training periods (non-acute samples), and from individuals who developed upper respiratory tract infection during training (acute samples). We analysed the samples using a commercial multiplex respiratory panel comprising 33 bacterial, viral and fungal targets. We used random effects tobit models to compare cycle threshold (Ct) value distributions from non-acute and acute samples.

**Results:**

We analysed 341 non-acute and 145 acute swab samples from 274 participants. *Haemophilus influenzae* type B was the most commonly detected microbe (77.4% of non-acute and 64.8% of acute samples). In acute samples, nine specific microbe pairs were detected more frequently than expected by chance. Regression models indicated significantly lower microbial load in non-acute relative to acute samples for *H. influenzae* non-type B, *Streptococcus pneumoniae* and rhinovirus, although it was not possible to identify a Ct-value threshold indicating causal etiology for any of these organisms.

**Conclusions:**

Semi-quantitative measures of microbial concentration did not reliably differentiate between illness and asymptomatic colonization, suggesting that clinical symptoms may not always be directly related to microbial load for common respiratory infections.

**Electronic supplementary material:**

The online version of this article (10.1186/s12879-018-3358-4) contains supplementary material, which is available to authorized users.

## Background

Multiplex polymerase chain reaction (PCR)-based diagnostic techniques allow rapid, simultaneous identification of a broad range of respiratory pathogens [1]. Compared to classical microbiological diagnostic methods, PCR-based assays offer higher sensitivity, specificity, and reproducibility [2]. However, the high sensitivity of multiplex PCR diagnostics does not directly translate into clinical utility, because such assays do not distinguish between viable and dead organisms, or acute infection and asymptomatic colonisation [2]. In the clinical setting, the etiological agent is seldom identified and unspecific respiratory symptoms are often treated empirically [3].

Although the quantification of microbial load may vary depending on the presence of co-infections, specimen type, sampling technique, or timing of sampling, quantitative or semi-quantitative microbial load data from real-time PCR assays may help define organism densities that are consistent with colonization or infection and distinguish between symptomatic and asymptomatic states [4]. In this study, we assessed whether semi-quantitative microbial load availab from real-time PCR assays can differentiate between symptomatic and asymptomatic states for common respiratory agents in a cohort of basic military trainees at two Royal Thai Army barracks.

## Methods

### Study settings and participants

Details of the study setting and procedures have been described previously [5]. Briefly, participants were recruited from six consecutive cohorts of basic military trainees at two Royal Thai Army barracks between May 2014 and July 2015. Trainees entered the camps for a 10-week training period at the start of May and November each year. Individuals aged ≥18 years entering one of the two army barracks involved in the study were eligible for enrolment. Suspected tuberculosis cases or individuals with immune deficiencies, such as acquired immune deficiency syndrome, leukemia or lymphoma, were excluded.

Throat and anterior nasal swab samples were collected using stiff synthetic swabs by trained study staff at the start and end of each training period (non-acute samples) and were placed in viral transport media (Universal Transport Medium C330; Copan Diagnostics) and stored at − 20 °C until time of transfer to the Armed Forces Research Institute of Medical Sciences for further testing. In addition, enrolled participants were asked to consult the camp’s medical unit if they experienced respiratory symptoms during the training period. Medical staff took a history, conducted a medical exam, and recorded symptoms of upper respiratory illness (URI) or influenza-like illness (ILI). URI was defined as an illness with at least two of the following: (i) runny nose or sneezing; (ii) nasal congestion; (iii) sore throat, hoarseness or difficulty swallowing; (iv) cough; (v) swollen or tender glands in the neck; and (vi) fever (oral temperature > 38 °C). ILI was defined as a respiratory illness with acute onset presenting with fever and cough or sore throat. Throat and nasal swab samples were collected on average 1.8 days after symptom onset from individuals who developed URI or ILI during the 10-week follow-up (acute samples).

### Laboratory investigations

Specimens from two of the six cohorts (total number of individuals = 274) were tested using a commercial multiplex real-time PCR assay comprising 33 bacterial, viral and fungal targets according to the manufacturer’s instructions (FTD33 kit, Fast Track Diagnostics, Esch-sur-Alzette, Luxembourg). These two cohorts were selected because they underwent concurrent routine environmental sampling of air and surfaces within the barracks, which were then similarly tested using the FTD33 kit (data not shown). Multiplex testing of specimens from the remaining cohorts was not done due to resource constraints. A cycle threshold (Ct) value below the detection limit of the assay (< 33) was considered a positive result.

### Statistical analyses

Non-acute samples collected at the end of the training period from participants who experienced an acute episode during follow-up were excluded from the analysis, as the Ct-value might reflect post-infectious shedding. We used the McNemar test to determine whether target-specific frequencies were significantly different in non-acute baseline samples and acute samples. In addition, we computed the chi-square (χ^2^) or Fisher’s exact test (for expected values < 5) to assess whether co-detection of specific microbe pairs occurred more frequently than expected by chance in non-acute baseline or acute samples. To account for data censoring at Ct-value = 33, random effects tobit regression models were used to compare Ct-value distributions from non-acute and acute samples, or Ct-value distributions from samples containing a single or multiple organisms. In addition, we used the Kruskal-Wallis test to compare the median delay between illness onset and sample collection between samples containing one or multiple organisms.

All analyses were conducted using Stata 12 software (Stata Corporation).

### Ethics, consent and permissions

The study was approved by the Institutional Review Boards of the Royal Thai Army in Bangkok, Thailand, the Walter Reed Army Institute of Research and the London School of Hygiene & Tropical Medicine. All participants provided written informed consent. The investigators have adhered to the policies for protection of human subjects as prescribed in Army Regulation 70–25.

## Results

### Microbe frequencies

We analyzed a total of 312 non-acute swab samples collected from 211 recruits at the start (*n* = 210) or end (*n* = 102) of the training period, and 145 acute specimens from 137 individuals who developed one or more URI episodes during follow-up. Of 33 targets contained in the respiratory panel, 19 were detected in at least one specimen (Table 1). Viruses were detected in 13.8% (43/312) and bacteria in 93.3% (291/312) of non-acute samples. Among acute samples, viruses were detected in 44.1% (64/145) and bacteria in 94.5% (137/145) of specimens.

**Table 1:** Median cycle threshold (Ct) values and interquartile range (IQR) for microbes identified in throat and nasal swab samples. Non-acute samples were collected from Royal Thai Army barracks trainees at the start (R0) and at the end (RF) of each 10-week training period. Acute samples were collected from trainees who developed acute upper respiratory tract infection during follow-up. Percentages indicate the proportion of collected samples positive for each organism

	Non-acute (*n* = 312)						Acute (*n* = 145)			*p*-value^a^
	R0 (*n* = 210)			RF (*n* = 102)						
	% (n)	Median Ct	IQR	% (n)	Median Ct	IQR	% (n)	Median Ct	IQR	
Bacteria										
*H. influenzae B*	83.8 (176)	28.3	26.5–29.6	65.7 (67)	29.7	28.6–31.4	64.8 (94)	29.4	28.0–31.3	0.297
*H. influenzae non-B*	31.9 (67)	30.2	28.4–31.1	73.5 (75)	24.9	22.1–27.1	56.6 (82)	26.7	22.0–29.0	< 0.001
*S. pneumoniae*	8.1 (17)	29.6	27.7–30.7	34.3 (35)	28.0	26.1–31.0	15.2 (22)	27.1	26.5–29.2	0.706
*K. pneumoniae*	14.3 (30)	31.2	28.3–32.7	9.8 (10)	29.0	26.8–32.0	15.9 (23)	31.4	29.7–32.3	0.706
*S. aureus*	10.5 (22)	31.3	30.4–32.5	9.8 (10)	31.4	31.1–32.2	6.2 (9)	31.1	30.6–31.6	0.739
*M. catarrhalis*	2.4 (5)	30.6	27.7–31.5	3.9 (4)	32.6	32.2–32.8	6.2 (9)	29.7	29.5–31.5	0.564
*L. pneumophila*	2.4 (5)	32.1	31.4–32.4	2.0 (2)	31.3	30.3–32.4	2.8 (4)	32.5	31.2–32.8	0.317
Viruses										
Rhinovirus	1.9 (4)	30.6	28.6–31.8	15.7 (16)	30.2	27.2–31.7	26.9 (39)	28.9	26.9–31.4	< 0.001
Adenovirus	0.5 (1)	27.2	27.2–27.2	9.8 (10)	30.5	29.6–31.4	2.8 (4)	29.3	27.0–31.0	0.083
Influenza B	0 (0)	–	–	0 (0)	–	–	9.7 (14)	25.4	23.6–31.2	–
Coronavirus 229	1 (2)	23.1	18.2–28.0	2.0 (2)	29.3	27.2–31.5	4.8 (7)	26.9	25.2–31.3	0.034
Enterovirus	0.5 (1)	30.7	30.7–30.7	4.9 (5)	30.2	30.1–30.3	0 (0)	–	–	–
Coronavirus 63	1 (2)	28.5	24.5–32.4	1.0 (1)	30.0	30.0–30.0	1.4 (2)	22.6	21.9–23.3	0.157
Coronavirus HKU	0.5 (1)	30.0	30.0–30.0	0 (0)	–	–	2.1 (3)	32.2	31.6–32.9	–
Parainfluenza 2	0.5 (1)	17.4	17.4–17.4	1.0 (1)	25.3	25.3–25.3	1.4 (2)	23.3	20.1–26.5	0.564
Parainfluenza 4	0.5 (1)	32.0	32.0–32.0	0 (0)	–	–	1.4 (2)	30.3	30.0–30.6	0.317
Coronavirus 43	0.5 (1)	28.1	28.1–28.1	0 (0)	–	–	0.7 (1)	32.6	32.6–32.6	–
Bocavirus	0.5 (1)	17.4	17.4–17.4	0 (0)	–	–	0 (0)	–	–	–
Influenza A	0 (0)	–	–	0 (0)	–	–	0.7 (1)	29.9	29.9–29.9	–

^a^*p*-value for difference in proportion of positive samples in non-acute samples collected at the start (R0) of the training period or acute samples from individuals who developed URI during follow-up, as computed by McNemar test

*Haemophilus influenzae* type B (Hi-B) was the most commonly detected microbe (77.9% of non-acute and 64.8% of acute samples). Other frequently detected bacteria included non-type B *Haemophilus influenzae* (Hi-nonB), *Streptococcus pneumoniae*, and *Klebsiella pneumoniae* (Table 1). Rhinovirus was the most prevalent virus, detected in 6.4% of non-acute and 26.9% of acute samples. All other viruses were detected in < 10% of collected specimens (Table 1).

Hi-nonB, rhinovirus, and coronavirus 229 were detected significantly less frequently in non-acute samples collected at the start of the training period than acute samples (*p*-values < 0.05) (Table 1). Influenza B was identified in none of the non-acute, but 9.7% of acute specimens.

### Frequency of microbe co-detection

**Fig. 1:**
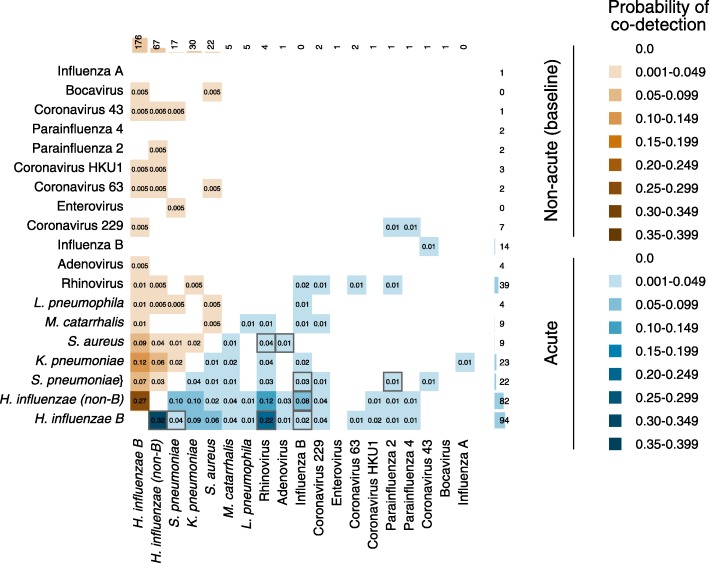
Pairwise probabilities of co-detection of bacterial and viral agents. Probabilities indicate the percentage of non-acute baseline (orange shading; *n* = 210) or acute samples (blue shading; *n* = 145) positive with each microbe pair. Bar charts on the upper or right hand side indicate number of non-acute baseline or acute samples positive for each microbe, respectively. Dark box outlines indicate microbe pairs detected more frequently than expected by chance, as assessed by χ2- or Fisher’s exact test (see also Table 3)

Multiple microbes were detected in 47.1% (99/210) of non-acute samples collected at the start of the training period. Co-detection of multiple organisms was significantly higher in both non-acute samples taken at the end of the training period (77.5%) and acute specimens (71.7%) (*p*-values < 0.001; Table 2). Among acute samples, 9 specific organism pairs were co-detected more frequently than expected by chance (*p*-values < 0.05) (Table 3; Fig. 1). Hi-B was identified together with Hi-nonB or rhinovirus in 32.4% (47/145) and 22.1% (32/145) of acute samples, respectively. Co-detection of influenza B virus and Hi-nonB occurred in 8.3% (12/145) of acute samples, while the remaining organism pairs were found in < 5% of acute specimens (Table 3). No microbe pair occurred more frequently than expected by chance among non-acute baseline samples.

**Table 2:** Frequency of single and multiple infections. Non-acute specimens were collected at the start (R0) or end (RF) of the training period. Acute samples were collected from individuals who developed an acute upper respiratory tract infection during follow-up. Percentages indicate proportion of specimens in which no microbe, a single, or multiple microbes were detected

	Non-acute		Acute	Total
	R0 (*n* = 210)	RF (*n*= 102)	(*n* = 145)	(*n*= 457)
Negative for all microbes	5.2% (11)	6.9% (7)	4.1% (6)	5.3% (24)
1 microbe detected	47.6% (100)	15.7% (16)	24.1% (35)	33.0% (151)
> 1 microbe detected	47.1% (99)	77.5% (79)	71.7% (104)	61.7% (282)

**Table 3:** Frequency of co-detections with selected organism pairs among acute samples. Acute specimens were collected from individuals who developed upper respiratory tract infection during follow-up (*n* = 145)

Microbe 1	%(n) detected^a^	Microbe 2	%(n) detected^a^	%(n) co-detected^b^	*p*-value^c^
*H. influenzae B*	64.8 (94)	*H. influenzae non-B*	56.6 (82)	32.4 (47)	0.031
*H. influenzae B*	64.8 (94)	Rhinovirus	26.9 (39)	22.1 (32)	0.008
Influenza B	9.7 (14)	*H. influenzae non-B*	56.6 (82)	8.3 (12)	0.024*
*H. influenzae B*	64.8 (94)	*S. pneumoniae*	15.2 (22)	4.1 (6)	< 0.001
*S. aureus*	6.2 (9)	Rhinovirus	26.9 (39)	4.1 (6)	0.012*
*S. pneumoniae*	15.2 (22)	Influenza B	9.7 (14)	3.4 (5)	0.04*
*H. influenzae B*	64.8 (94)	Influenza B	9.7 (14)	2.1 (3)	0.001*
*S. pneumoniae*	15.2 (22)	Parainfluenza 2	1.4 (2)	1.4 (2)	0.022*
*S. aureus*	6.2 (9)	Human adenovirus	2.8 (4)	1.4 (2)	0.019*

^a^Percentages indicate proportion of acute samples where each microbe was identified

^b^Percentages indicate proportion of acute samples where each microbe pair was co-detected

^c^*p*-value computed by χ^2^- or Fisher’s exact (*) test, indicating that co-detection of each of the listed microbe pairs occurred more frequently than expected by chance

### Microbial load

**Fig. 2:**
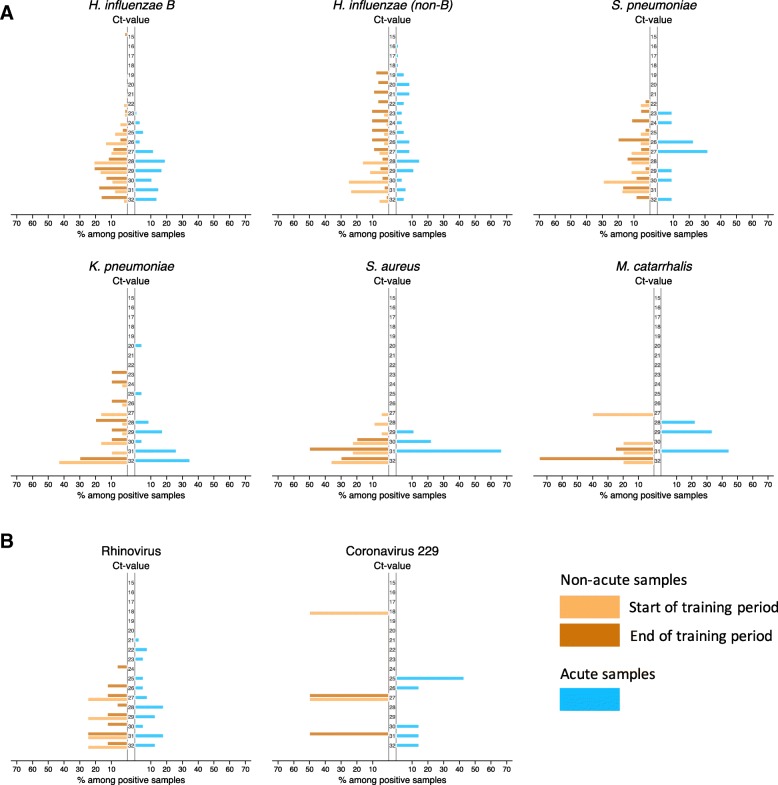
Cycle threshold value distribution in non-acute and acute samples. Ct-value distribution for selected **a** bacteria and **b** viruses detected in non-acute samples collected at the start or end of the training period (orange bars) or acute samples from individuals experiencing an upper respiratory tract infection during follow-up (blue bars). A Ct-value of < 33 was considered a positive result

**Fig. 3:**
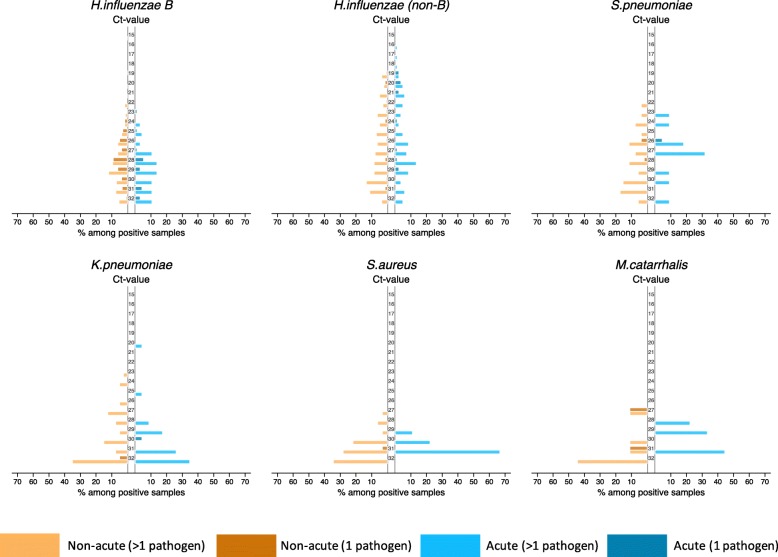
Cycle threshold value distribution in samples containing one or multiple microbes. Ct-value distributions for selected bacteria detected in non-acute samples collected at the start or end of the training period (orange bars) and acute samples (blue bars) containing a single or multiple microbes. A Ct-value of < 33 was considered a positive result

Overall, there was a substantial overlap in Ct-value distributions from non-acute samples collected at the start or end of the training period and acute samples collected from symptomatic individuals during follow-up (Fig. 2). This was the case even when considering only samples where a single organism was identified (Fig. 3).

For Hi-nonB and *S. pneumoniae*, our tobit regression models indicated significantly lower microbial load in non-acute baseline compared to acute samples (*p*-values < 0.05) (Table 4). For Hi-nonB, a coefficient of 5.56 represents a 5.56 higher average Ct-value in non-acute baseline samples compared to acute specimens, which corresponds to an approximately 47-fold lower microbial load in non-acute compared to acute samples. For *S. pneumoniae*, the average microbial load was 8.2-fold lower in non-acute baseline samples compared to acute specimens. Our analysis also indicated a significantly lower average rhinovirus load in non-acute samples collected either at the start or at the end of the training period compared to acute samples (*p*-values < 0.05) (Table 4). This was in contrast with Hi-B, for which regression analysis indicated a 4.7-fold higher average microbial load in non-acute baseline samples compared to acute samples (*p*-value < 0.001) (Table 4). For Hi-non B and *S. pneumoniae*, there was a 7.7-fold or 19.4-fold increase in average microbial load in non-acute samples collected at the end of follow-up compared to acute samples collected during an URI episode, respectively (*p*-values ≤0.002).

**Table 4:** Tobit regression analysis. Tobit regression model assessing differences in Ct-value distributions in non-acute samples collected at the start (R0) or end (RF) of the training period and acute samples collected from symptomatic individuals during follow-up. Acute samples are used as a reference

Bacteria	Sample type	Coefficient	95%CI	*p*-value
*H. influenzae B*	R0	−2.22	−2.94; −1.49	< 0.001
	RF	0.11	−0.80; 1.02	0.809
*H. influenza non-B*	R0	5.56	4.05; 7.08	< 0.001
	RF	−2.95	−4.62; −1.27	0.001
*S. pneumoniae*	R0	3.04	0.41; 5.67	0.024
	RF	−4.28	−7.00; −1.56	0.002
*K. pneumoniae*	R0	0.52	−1.01; 2.05	0.506
	RF	1.45	−0.55; 3.45	0.155
*S. aureus*	R0	−0.98	−2.43; 0.47	0.186
	RF	−0.74	−2.47; 0.99	0.401
Viruses				
Rhinovirus	R0	10.04	6.49; 13.58	< 0.001
	RF	2.83	0.39; 5.27	0.023

### Time to sample collection

There was no significant difference in delay between symptom onset and specimen collection in acute samples containing one (median delay: 2 days; interquartile range (IQR): 1–3) or more (median delay: 2 days; IQR: 1–3) organisms (*p*-value = 0.536). Six acute specimens were negative for all agents tested (median delay: 0.5 days; IQR: 0–1). Thus, sampling delay is unlikely to account for any observed differences in Ct-value distributions.

## Discussion

### Microbe frequencies

We analyzed the patterns of infection with common respiratory agents in a well-defined population of military recruits. The use of highly sensitive multiplex PCR diagnostics allowed an accurate characterization of the spectrum of organisms contained in non-acute and acute samples.

The data indicate co-circulation of several different viral agents, and high frequency of bacterial colonization in both non-acute and acute samples. Up to one third of respiratory illness cases among army personnel are reportedly caused by viral or bacterial infections [6]. The gathering of individuals from diverse geographic locations and the crowded living conditions increase the risk of microbe transmission in these settings [7]. Illnesses are usually self-limiting, although the emergence of highly virulent strains can lead to high morbidity and mortality [8]. *Streptococcus* bacteria, adenoviruses, coronaviruses and influenza are among the most widely distributed microbes in the military environment, and are implicated in > 50% of febrile illness cases reported at military medical facilities [6]. We identified each of these organisms in one or more samples. For most of these microbes, overall detection frequencies were comparable in non-acute and acute samples, although influenza B and coronavirus 229 were more commonly identified among acute specimens. Other infectious agents commonly circulating among military personnel include *H. influenzae*, rhinovirus, and, to a lesser extent, parainfluenza, RSV, and *L. pneumophila*, although their presence does not necessarily imply the occurrence of clinical symptoms [9–11]. *H. influenzae* and rhinoviruses were the most frequently detected organisms in our population in both non-acute and acute samples. We detected parainfluenza and *L. pneumophila*, but we did not find RSV in any of our samples.

### Clinical relevance

For individuals developing URI during follow-up, illness etiology could not be unequivocally determined. Among acute samples, Hi-B was the most frequently detected organism. It was the sole agent identified in 12% of acute specimens, while it was co-detected with other microbes in > 50% of acute samples. However, colonisation with Hi-B was also common among non-acute baseline samples, where it was detected alone or in combination with other microbes in 40.5% and 43.3% of specimens, respectively.

For organisms rarely detected among asymptomatic individuals but frequently found in acute samples, a causal association may be more likely. For instance, influenza B was detected in none of the non-acute, but 9.7% of acute samples. Similarly, the proportion of both Hi-nonB- and rhinovirus-positive samples was significantly lower among non-acute specimens collected at baseline compared to acute samples. However, > 85% of acute samples positive for Hi-non B, rhinovirus or influenza B were also positive for one or more additional microbe, so that a causal association could not be determined. Some agents, such as Hi-non B or adenovirus, were most frequently detected in non-acute samples collected at the end of follow-up, possibly indicating post-infectious shedding or persistent infection at sub-clinical levels.

In the clinical setting, overlapping clinical presentations and poor capabilities to determine the etiology of respiratory illnesses often lead to inappropriate treatment with broad-spectrum antibiotics [12]. This might occur even more frequently in the military setting, where molecular diagnostic tools are usually inaccessible [6]. Since a considerable fraction of respiratory illnesses is caused by viruses, the unsubstantiated use of antibiotics is particularly problematic, because it can lead to negative health outcomes and promote the development of antimicrobial resistance [3]. Studies evaluating the impact of multiplex diagnostic procedures on patient management report inconsistent results. In the outpatient setting, access to rapid molecular diagnostic tools for respiratory pathogens significantly reduced antibiotic prescription rates for patients presenting with respiratory illness [13]. However, these findings were not confirmed in the hospital setting. PCR-based testing failed to reduce hospital admissions and duration of hospital stay in patients with acute respiratory infection [14, 15]. Although molecular diagnostic tools may help to differentiate bacterial and viral respiratory agents, it is unlikely that antibacterial treatment would be terminated based on the mere presence of viral agents in an acute respiratory sample, especially considering the high rates of bacterial co-infection [16].

### Microbial load

Quantitative or semi-quantitative diagnostic tools can potentially help define clinically significant pathogen densities, and have proven highly valuable to understand the dynamics of diarrheal disease [17] and to improve the management of gastrointestinal illnesses [18]. Among acute diarrhea patients, quantitative amplification of norovirus RNA from fecal samples can help determine pathogen load thresholds that effectively distinguish between causal association and sub-pathogenic carriage [19]. Similarly, rotavirus load correlates with disease severity among children with gastroenteritis [20]. Because of the crucial role of microbial replication in viral pathogenesis, the value of pathogen load quantitation could be most clearly established for gastrointestinal illnesses of viral etiology, although some evidence is available for bacterial infections as well. For instance, microbial load of enteropathogenic *E. coli* is significantly higher among children with diarrhea compared to control subjects, especially when enteropathogenic *E. coli* is the sole agent identified [21].

In this study, tobit regression indicated significantly lower microbial load in non-acute relative to acute samples for rhinovirus, HI-nonB, and *S. pneumoniae*. However, due to a substantial overlap in Ct-value distributions, it was not possible to identify a Ct-value threshold indicating causality for any of these organisms. Previous studies assessing the association of viral load with clinical symptoms of respiratory infections reported similar findings. Mean viral load for rhinovirus and six additional viruses was significantly higher in upper respiratory tract aspirates from children with pneumonia compared to healthy controls, but the overlap in viral load distribution was substantial [22]. In pediatric patients, high rhinovirus load was associated with the presence of lower respiratory tract symptoms [23, 24], but a threshold for clinical relevance could only be determined if rhinovirus was the sole agent identified [24]. Additional studies reported a correlation between microbial load and occurrence or severity of respiratory symptoms for RSV [25], bocavirus [26], and human metapneumovirus (HMPV) [27, 28], although these findings were inconsistent [29, 30] or conditional on the presence of the virus as a single microbe [31]. We did not detect any significant association between microbial load and clinical manifestations for viruses other than rhinovirus.

For both *H. influenzae* and *Streptococcus* species, previous studies reported a significant correlation of bacterial densities with clinical manifestations of disease [32]. In young patients with acute respiratory tract infection, *S. pneumoniae* load fluctuated with symptom incidence and resolution [33]. Among children hospitalized with pneumonia, median nasopharyngeal *S. pneumoniae* load was substantially higher compared to healthy controls [32]. Pneumococcal density was also associated with severity of symptoms [34] and increased duration of children’s hospital stay [35]. Similar associations were observed in pneumonic adults, although the correlation was not significant in this population [36].

The association between microbial load and clinical manifestations may depend on specific pathogen-host interactions. If pathogenesis is primarily related to microbial replication, a stronger correlation between microbial load and illness magnitude may be observed [37]. If clinical manifestations are largely attributable to host immune defences or bacterial toxins, the correlation with microbial load may not be obvious [37]. Temporal variations in microbial load may also play an important role if the quantity of nucleic acid is significantly more abundant at the time and location of pathology [30, 33]. In acute respiratory illness patients, high bacterial colonization densities are often associated with the presence of viral co-infections [38], and clinical manifestations may vary depending on specific co-infection patterns [39].

The ecology of respiratory pathogens is also likely to be influenced by the living conditions in military settings. Mixing of individuals from diverse backgrounds living in close-quarters with high levels of inter-personal contact increases the potential for introduction and spread of multiple microbes in this population, which could account for the broad range of organisms and co-detections in this study.

### Strengths and limitations

We analysed both non-acute and acute samples from a closely monitored population in a semi-closed, longitudinal setting. The study population was well-defined and relatively homogeneous with regards to demographics and living conditions. However, our findings may not be applicable to populations with different socio-demographic characteristics and populations outside the military environment, such as cohorts of children among whom the impact of respiratory infections may be greater.

The frequent co-detection of multiple respiratory agents and the failure to distinguish between viable and dead organisms, or microbes that colonize the host at sub-pathogenic levels, may prevent the unambiguous interpretation of test results [2]. A positive result may indicate illness aetiology, asymptomatic colonisation, post-infectious shedding, or an incipient infection. Therefore, Ct-values may not always be a reliable surrogate for infectious load.

Samples from only two out of six cohorts were tested by real-time PCR. Although there might be bias from seasonal effects, these are usually less pronounced in the tropics. Given the relatively low frequencies of viral detection, a larger sample size and a longer follow-up may have captured a more precise picture of infection patterns in this population. This study was also limited to the detection of organisms contained in the respiratory panel. We cannot exclude the presence of additional organisms in our specimens. In addition, the data were obtained from throat and nasal swab samples, but our findings may not apply to nasopharyngeal or sputum specimens. Finally, the quality and quantity of material obtained through nose and throat swabs may differ significantly among subjects, and the success of PCR-based methods also depends on the availability of intact genome sequences and the absence of random mutations.

## Conclusions

Overall, the multiplex respiratory panel provided a comprehensive characterization of the microbe spectrum contained in non-acute and acute respiratory samples collected among recruits. However, semi-quantitative assessment of microbial load could not reliably distinguish between symptomatic and asymptomatic samples. More research is warranted to compare new multiplex diagnostic techniques with traditional methods and evaluate their potential with regards to diagnostic accuracy [40] and clinical utility [16, 40] in the context of respiratory infections.

## Additional file

Additional file 1: Dataset. Title of data: Semi-quantitative microbial load in throat and nasal swab samples from Thai Army recruits. Description of data: Semi-quantitative microbial load in non-acute and acute throat and nasal swab samples from Thai Army recruits, determined using a commercial multiplex real-time PCR assay comprising 33 bacterial, viral and fungal targets; includes names, labels, and coding for individual variables. (XLS 311 kb)

## Abbreviations

PCR: Polymerase chain reaction URI Upper respiratory illness ILI Influenza-like illness FTD Fast Track Diagnostics Ct Cycle threshold Hi-B *Haemophilus influenzae* type B Hi-nonB non-type B *Haemophilus influenzae* IQR Interquartile range HMPV Human metapneumovirus
